# Differentiating Hypoglycemia Awareness Status from Hypoglycemia Experience in Tools for Measuring Impaired Awareness of Hypoglycemia

**DOI:** 10.1089/dia.2020.0034

**Published:** 2020-06-30

**Authors:** Eduardo Sepúlveda, Rui Poínhos, Gil Nata, Davide Carvalho, João Sérgio Neves, Daniela Seixas, Pratik Choudhary, Selene G. Vicente, Stephanie A. Amiel

**Affiliations:** ^1^ Centre for Psychology at University of Porto, Faculty of Psychology and Educational Sciences, University of Porto, Porto, Portugal.; ^2^ Department of Diabetes, School of Life Course Sciences, King's College London, London, United Kingdom.; ^3^ Faculty of Nutrition and Food Sciences, University of Porto, Porto, Portugal.; ^4^ Centre for Research and Intervention in Education and Centre for Psychology at University of Porto, University of Porto, Porto, Portugal.; ^5^ Department of Endocrinology, Diabetes and Metabolism, Centro Hospitalar São João, Porto, Portugal.; ^6^ Faculty of Medicine, University of Porto, Porto, Portugal.; ^7^ Instituto de Investigação e Inovação em Saúde, Universidade do Porto, Porto, Portugal.; ^8^ Unidade de Investigação Cardiovascular, Departamento de Cirurgia e Fisiologia, Faculty of Medicine, University of Porto, Porto, Portugal.; ^9^ Institute for Biomedical Imaging and Life Sciences, University of Coimbra, Coimbra, Portugal.; ^10^ King's College Hospital NHS Foundation Trust, London, United Kingdom.

**Keywords:** Type 1 diabetes, Severe hypoglycemia, Hypoglycemia awareness

## Abstract

***Background:*** Developing technologies in real-time continuous glucose monitoring (CGM) are successfully reducing severe hypoglycemia (SH) in trials and clinical practice. Their impact on impaired awareness of hypoglycemia, a major risk factor for SH, is uncertain.

***Methods:*** The present study examined two scales for assessing hypoglycemia awareness status, the Gold score and the eight-item Minimally Modified Clarke Hypoglycemia Survey (MMCHS), commonly used in trials of CGM, in Portuguese-speaking adults with type 1 diabetes and conducted an exploratory factor analysis on MMCHS.

***Results:*** A bifactorial structure in MMCHS was revealed, with a clear distinction between items that measure SH experience and those that measure hypoglycemia awareness status. The latter is associated with the same risk for SH as the Gold score.

***Conclusions:*** We conclude that improvement in awareness scores by the MMCHS may reflect only a reduction in SH with no restoration of endogenous awareness, making the current literature consistent in evidence that CGM does not improve endogenous awareness and nonsensor supported protection from SH. This has implications for risk of SH when CGM is not being worn.

## Background

Hypoglycemia remains a major concern for people with diabetes treated with insulin or insulin secretagogues and their families.^1–3^ Impaired awareness of hypoglycemia (IAH) is a major risk factor for severe hypoglycemia (SH),^4^ episodes in which hypoglycemia-related cognitive impairment renders people unable to self-treat.^5,6^ SH can have severe consequences, including death.^5^ New technologies in insulin delivery, particularly in real-time continuous glucose monitoring (CGM), have been demonstrated significantly to reduce risk of SH, providing external information about and warnings of falling circulating glucose concentrations. In theory, reduced exposure to hypoglycemia should be associated with restoration of subjective awareness of occasional episodes, but whether CGM use is associated with restored awareness remains controversial.^7–12^ Current evidence suggests that the protection against SH is provided only when the devices are being worn.

Awareness of hypoglycemia is commonly measured using one of two scales, both developed in the 1990s. The Gold score^13^ asks one question, in which the person with diabetes rates on a linear scale the degree to which they know when a hypoglycemic episode is commencing (1 = always aware, 7 = never aware). The Clarke score^14^ poses eight questions (Table 1), answers to which are scored as indicating awareness (A) or reduced awareness (R). The Clarke score for R also ranges from 0 to 7, two of the questions being combined to give one answer. The original score has been minimally modified, to take account of current definitions of SH, into the Minimally Modified Clarke Hypoglycemia Survey (MMCHS).^15^ In both Gold score and MMCHS, a score of 4 or higher is interpreted as IAH and is associated with a six-fold increased risk of SH.

**Table 1.** Minimally Modified Clarke Hypoglycemia Survey Principal Component Analysis

KMO		0.69	
Bartlett (*P*)		**<0.001**	
Factor		F1	F2
Eigenvalue		1.97	1.56
Variance (%)		28.19	22.22
Correlation with the principal component	Item 1 (I always/sometimes/no longer have symptoms when my blood sugar is low.)	**0.66**	0.28
	Item 2 (Have you lost some of the symptoms that used to occur when your blood sugar was low?)	**0.38**	0.36
	Item 3 (In the past 6 months, how often have you had hypoglycemic episodes, where you might feel confused, disorientated, or lethargic and were unable to treat yourself?)	0.08	**0.84**
	Item 4 (In the past year, how often have you had hypoglycemic episodes, where you were unconscious or had a seizure and needed glucagon or intravenous glucose?)	0.07	**0.80**
	Item 5/6 (How often in the last month have you had readings <3.5 mmol/L [<63 mg/dL] with symptoms?/How often in the last month have you had readings <3.5 mmol/L [<63 mg/dL] without any symptoms?)	**0.69**	0.03
	Item 7 (How low does your blood sugar need to go before you feel symptoms?)	**0.79**	0.02
	Item 8 (To what extent can you tell by your symptoms that your blood sugar is low?)	**0.54**	0.09

Significant values and highest loadings are indicated in bold type.

KMO, Kaiser-Meyer-Olkin.

We measured hypoglycemia awareness in a clinic sample of adults with type 1 diabetes (T1D) using both Gold score and MMCHS and investigated the latter using an exploratory factor analysis in a secondary/tertiary diabetes service in Portugal.

## Methods

### Participants

The study was conducted from September 2016 to December 2018. Adults with T1D attending the diabetes clinic of the Centro Hospitalar São João, Portugal, were invited to participate. Eligible participants were aged ≥18 years, with ≥12 months T1D, adequate visual and auditory acuity, able to understand written and spoken European Portuguese, with available medical records. Exclusion criteria included pregnancy, scores above 14 in either of the subscales of the Hospital Anxiety and Depression Scale,^16^ neurological diseases, and other medical conditions that might compromise their participation in the study. The study was approved by the Ethics Committee for Health of Centro Hospitalar São João.

### Procedures

The Gold score and MMCHS were translated from English into European Portuguese by two independent bilingual translators, a synthesis of the two versions created, assessed by a panel of experts, and used by participants with T1D in a cognitive debriefing to check the adequacy of the instructions and the structure and adequacy of the items. A back translation was performed by a third bilingual translator.

Eligible participants were approached in the clinic, asked if they might be interested in the research, offered an information sheet and the opportunity to ask questions about the research. Consent to proceed was obtained in writing, after which participants completed the two scores in the presence of one of the investigators (E.S.) during the clinic attendance. Demographic and clinical data were obtained from hospital records. IAH was considered present if the Gold score was ≥4. Hypoglycemia awareness status was also calculated from the MMCHS, with a score of ≥4 considered IAH. A score of R in either item 3 and/or 4 of the MMCHS was taken as evidence of at least one SH in the 6–12 months before study. Annual rates of SH were determined using item 4 of the MMCHS (SH_12m_).

### Statistical analyses

IBM SPSS version 24.0 for Windows software was used for the analysis. Descriptive statistics are presented as mean ± standard deviation or absolute (*n*) and relative (%) frequencies. We compared our sample with the overall clinic sample in terms of age (using an independent samples *t*-test) and regarding sex (using chi-square [χ^2^] test with Yates continuity correction). Independent samples *t*-tests were used (i) to assess differences between participants with T1D with and without IAH by Gold score or by factor 1 of the MMCHS in terms of experience of SH (SH rates) and (ii) to evaluate differences between those with and without IAH by Gold score in terms of age, diabetes duration, and HbA1c. The χ^2^ test with Yates continuity correction was used to assess the independency between hypoglycemia awareness status measured with the Gold score and (i) sex and (ii) SH in the 6–12 months before study. Gold score and MMCHS definitions of IAH (MMCHS total score and five-item factor 1 of the MMCHS) were correlated using Pearson's correlation coefficient. Exploratory factor analysis of the MMCHS was performed through principal components analysis, with Varimax rotation; each item was considered to belong to the factor, in which it had the highest loading, with a minimum value of 0.38.^17^ The Kaiser-Meyer-Olkin measure of sampling adequacy and the Bartlett's test were used to validate the factor analysis model.^18^ An eigenvalue of >1.0 was taken as evidence of robust factorial allocation.^19,20^ Internal consistency reliability was measured using Cronbach's alpha coefficient or Pearson's correlation for factors of less than three items.^19^ Relationships were considered to be statistically significant at *P* < 0.05.

## Results

We recruited 190 adults with T1D. The mean diabetes duration and HbA1c were 20.10 ± 11.28 years and 8.07% ± 1.52%, respectively. None was using real-time CGM. When compared to the overall T1D attending the same clinic (*n* = 468), our sample did not differ regarding the percentage of females (51.58% vs. 46.79%, χ^2^ = 1.06, *P* = 0.304), but had lower age [38.24 ± 12.92 vs. 42.00 ± 14.23 years, *t* (654) = 3.15, *P* = 0.002].

By Gold score, 23.68% of participants were defined as having IAH. People with IAH by Gold score had higher annual rates of SH_12m_ than those with intact awareness [1.42 ± 3.24 vs. 0.29 ± 1.31, *t* (48.56) = −2.29, *P* = 0.026]. They had longer diabetes duration [23.33 ± 12.54 vs. 19.08 ± 10.70 years, *t* (186) = −2.23, *P* = 0.027], but did not differ in terms of age [40.71 ± 14.18 vs. 37.48 ± 12.46 years, *t* (188) = −1.47, *P* = 0.143]; HbA1c [8.00 ± 1.61 vs. 8.09 ± 1.49%, *t* (187) = 0.35, *P* = 0.728]; or percentage of females (60.00% vs. 48.97%, χ^2^ = 1.26, *P* = 0.261). At least one SH in the 6–12 months before the study was reported by 36.84%, with 51.11% reporting at least one such episode in the IAH group versus 32.41% in those with Gold score of less than 4 (χ^2^ = 4.39, *P* = 0.036).

By total MMCHS, a lower prevalence of IAH (14.29%) was found than with the Gold score, but the two measures were correlated (*r* = 0.56, *P* < 0.001).

The unforced exploratory factor analysis of MMCHS data (Table 1) identified two factors (eigenvalues >1.0). KMO and Bartlett's test values proved adequate (KMO = 0.69; Bartlett's test: *P* < 0.001). Factor 1 included items 1, 2, 5/6, 7, and 8 and we called this the Hypoglycemia Awareness Factor. Factor 2 included items 3 and 4 and we called this SH Experienced. Together, they explained 50.41% of the variance. Reliability analysis showed acceptable Cronbach's alpha (0.63) for factor 1, given the number of items. The removal of any item resulted in a lower value. The correlation between the two items of the second factor was significant (*r* = 0.43, *P* < 0.001).

In a linear regression predicting factor 1 of MMCHS's score based on Gold score, the value for a Gold score of 4 was given by: −0.14 + 0.49 × 4 = 1.82.

Using a factor 1 score of 2 or more to define IAH by MMCHS gave a prevalence of IAH for 29.10%, with a rate of SH_12m_ of 1.22 ± 2.96, compared to a rate of 0.29 ± 1.36 in those scoring <2 [*t* (63.55) = −2.23, *P* = 0.029]. The correlation between the five-item factor 1 of the MMCHS and the Gold score was statistically significant (*r* = 0.60, *P* < 0.001).

## Discussion

The Gold score and MMCHS are well validated systems for scoring awareness of hypoglycemia. IAH, diagnosed by a score of 4 or more on either, predicts high risk for SH.^13,14,21^ Maintenance or restoration of awareness of hypoglycemia is therefore an important outcome for trials of new diabetes therapies and technologies. This study is the first to empirically validate a bifactorial structure of the MMCHS, with one factor driven by exposure to SH and the other related to hypoglycemia awareness status alone. This has implications for studies seeking to assess new interventions for their ability to maintain or restore hypoglycemia awareness, as awareness status assessed by the total MMCHS as originally described may be altered by a change in SH experience alone.

IAH and SH are closely related, but they are not the same. Technologies such as real-time CGM have a major impact on SH rate.^7–9^ In many guidelines, problematic hypoglycemia is an evidence-based indication for CGM.^22,23^ Although use of CGM is associated with reduced SH and decreased time below range, and reduced exposure to hypoglycemia should in theory improve awareness,^24–26^ only one study has described improved awareness after CGM, using total MMCHS.^12^ The result may have been driven by the reduction in SH achieved. Restoration of subjective awareness of hypoglycemia would imply a degree of protection against further SH and possible benefit during intervals when the CGM is not being used or is not functioning. We are not aware of any evidence to suggest that this might be the case.

In our study, prevalence of IAH by Gold score was directly comparable to other studies of adults with T1D,^2,4^ and the increased risk of SH in people with IAH likewise comparable to published data.^2,21,27^ The longer diabetes duration of those with IAH by Gold score in our study has also been reported elsewhere.^4^ The total MMCHS showed significant correlation with the Gold score. This is important to note, as the study was the first to use both the scores in European Portuguese. The full MMCHS includes eight items, with two scored together, giving a maximum score of 7. Our five-item hypoglycemia awareness factor had an acceptable Cronbach's alpha for a factor of this number of items,^28^ correlated with the Gold score and carried the same association with SH. The hypoglycemia awareness factor corresponds to factor 1 of MMCHS with a cutoff of ≥2 for IAH.

Limitations of our study are that SH rate was assessed only by the MMCHS questionnaire, which does not fully quantify an annual rate of SH, and we did not verify awareness status with an experimentally induced episode. The Gold score and MMCHS derive from subjective responses about accidental hypoglycemia, so these limitations should not impact on the outcome of the factor analysis of the latter.

We suggest that, while the total MMCHS gives an excellent picture of overall problematic hypoglycemia, the impact of any therapy on endogenous hypoglycemia awareness status may be better judged by the Gold score and/or by the score of the five-item hypoglycemia awareness factor of the MMCHS score alone. It is an important distinction, perhaps particularly in the assessment of new technologies, as restoration of endogenous awareness of hypoglycemia should provide some protection from SH in times when technology is not being worn. The Gold score remains a useful clinical tool for assessing awareness of hypoglycemia independent of experience of severe episodes, but the five-item factor of MMCHS should prove a useful adjunct, particularly in research, because it includes some more quantitative items and is thus less purely subjective and it might replace the full MMCHS when endogenous awareness of hypoglycemia is the focus of interest.

## Duality of Interest

S.A.A. has served on advisory boards for Medtronic, Abbott, and Novo Nordisk. P.C. has received personal fees from Novo Nordisk, Sanofi, Lilly, Medtronic, Abbott, Novartis, Insulet, and Dexcom. The other authors have no duality to declare.
